# Transcranial direct current stimulation induces long-term potentiation-like plasticity in the human visual cortex

**DOI:** 10.1038/s41398-020-01134-4

**Published:** 2021-01-04

**Authors:** Lukas Frase, Lydia Mertens, Arno Krahl, Kriti Bhatia, Bernd Feige, Sven P. Heinrich, Stefan Vestring, Christoph Nissen, Katharina Domschke, Michael Bach, Claus Normann

**Affiliations:** 1 Department of Psychiatry and Psychotherapy, Medical Center, University of Freiburg—Faculty of Medicine, University of Freiburg, Freiburg, Germany; 2 Eye Center, Medical Center, University of Freiburg—Faculty of Medicine, University of Freiburg, Freiburg, Germany; 3 University Hospital of Psychiatry and Psychotherapy, University of Bern, Bern, Switzerland; 4 Center for Basics in Neuromodulation, Freiburg, Germany

**Keywords:** Depression, Learning and memory

## Abstract

Transcranial direct current stimulation (tDCS) is increasingly used as a form of noninvasive brain stimulation to treat psychiatric disorders; however, its mechanism of action remains unclear. Prolonged visual stimulation (PVS) can enhance evoked EEG potentials (visually evoked potentials, VEPs) and has been proposed as a tool to examine long-term potentiation (LTP) in humans. The objective of the current study was to induce and analyze VEP plasticity and examine whether tDCS could either modulate or mimic plasticity changes induced by PVS. Thirty-eight healthy participants received tDCS, PVS, either treatment combined or neither treatment, with stimulation sessions being separated by one week. One session consisted of a baseline VEP measurement, one stimulation block, and six test VEP measurements. For PVS, a checkerboard reversal pattern was presented, and for tDCS, a constant current of 1 mA was applied via each bioccipital anodal target electrode for 10 min (Fig. S1). Both stimulation types decreased amplitudes of C1 compared to no stimulation (*F* = 10.1; *p* = 0.002) and led to a significantly smaller increase (PVS) or even decrease (tDCS) in N1 compared to no stimulation (*F* = 4.7; *p* = 0.034). While all stimulation types increased P1 amplitudes, the linear mixed effects model did not detect a significant difference between active stimulation and no stimulation. Combined stimulation induced sustained plastic modulation of C1 and N1 but with a smaller effect size than what would be expected for an additive effect. The results demonstrate that tDCS can directly induce LTP-like plasticity in the human cortex and suggest a mechanism of action of tDCS relying on the restoration of dysregulated synaptic plasticity in psychiatric disorders such as depression and schizophrenia.

## Introduction

Long-term potentiation (LTP) is considered to be one of the main mechanisms underlying brain plasticity, learning and memory^1^. In animal models, LTP of synaptic transmission in the hippocampus has been conceptualized as a persistent strengthening of synapses via rapid repetitive or paired associative neuronal inputs^2^. In humans, low-frequency median nerve stimulation paired with transcranial magnetic stimulation of the motor cortex has been demonstrated to induce LTP-like plasticity in motor-evoked potentials^3^. In other brain areas, research has been limited by the lack of feasible experimental procedures able to induce and analyze potential LTP induction.

Recently, analyzing specific electroencephalogram (EEG) responses induced by sensory stimulation has been proposed as a tool to examine LTP in various brain areas. Repeated high-frequency presentations of visual stimuli (prolonged visual stimulation, PVS) persistently enhanced visually evoked potentials (VEP)^4,5^. VEPs typically comprise several defined amplitude deflections: an early negativity at ~75 ms (C1, sometimes referred to as N75), followed by a positive peak at ~100 ms (P1/P100) and another negativity at 145 ms (N1/N145), which some authors describe as having two parts (N1a, N1b)^4^. While subsequent EEG response patterns beyond 300 ms (such as the P300) are modulated by complex cognitive processes, these early EEG responses are mainly related to local neural activity in the visual cortex^6^. In the original experiment by Teyler et al.^4^, only the N1b component was significantly potentiated by PVS. Later studies show increases in P1, as well as N1, amplitudes^5,7,8^. In rodent experiments, VEP plasticity has been shown to rely on thalamocortical LTP and to share many common properties with the canonical form of LTP in hippocampal brain slices, such as input specificity, cooperativity and persistence; moreover, VEP plasticity requires the activation of *N*-methyl-d-aspartate (NMDA) receptors and the delivery of α-amino-3-hydroxy-5-methyl-4-isoxazolepropionic acid (AMPA) receptors containing the GluR1 subunit^9,10^. VEP plasticity has since been established as a tool to examine patterns of disturbed neural plasticity in mental disorders. Decreased VEP plasticity in the visual cortex has been described in patients suffering from schizophrenia^8,11^, depression^5^, and bipolar disorder^7,12^.

In these conditions, noninvasive brain stimulation in distinct cortical areas is increasingly applied as a biological treatment approach, potentially acting by normalizing neural plasticity^13^. One of these techniques, transcranial direct current stimulation (tDCS), is currently being evaluated for the treatment of depression^14^, neurological rehabilitation^15^, and cognitive performance^16^. It is still largely unclear how tDCS affects brain plasticity on a cellular level. The effects of tDCS might be due to changes in the membrane excitability of cortical neurons^17^ or due to metaplastic effects^18,19^ and might result in an LTP-like phenomenon^20,21^, at least in the motor cortex. In the visual cortex of healthy participants, tDCS over the occipital cortex modulated mainly the C1 amplitudes of single VEPs^18,22^. Whether tDCS modulates P1 remains unclear, with some studies failing to find effects^22^ and others describing an increase in the P1 amplitude following anodal tDCS^18,23^. Recently, researchers have renewed the discussion about the fundamental assumption whether currents applied by standard tDCS protocols are capable of reaching the brain^24^.

The current study combined anodal tDCS over the occipital cortex with PVS. This study first implemented VEP plasticity as an established tool for the induction and analysis of LTP-like plasticity in the human brain and then examined whether tDCS could modulate or mimic VEP plasticity induced by PVS. A modulation of VEP plasticity by tDCS would add further evidence in support of the plasticity-enhancing effects of tDCS in humans and would aid in revealing the mechanism of action of tDCS in disorders that are associated with disturbed brain plasticity^5,16,25,26^.

## Material and methods

### Subjects

A total of 38 healthy participants (20 females, 18 males; age 24.2 ± 2.0 years, age range 21–31 years) were included in the analysis to be on par with studies demonstrating clear effects on VEP plasticity^5,12^. Seven additional participants were not included in the analysis: two participants suffered from a febrile infection that might have influenced VEP amplitudes, one participant was identified as being amblyopic after screening, and four participants did not complete the protocol due to technical issues. All participants underwent extensive screening by experienced psychiatrists to rule out any relevant mental or physical disorders or any tDCS-specific exclusion criteria^27^. All participants were free of any CNS-active medication, were right handed, were nonsmokers and did not consume any caffeine or alcohol during the study. Normal or corrected-to-normal visual acuity was confirmed by the Freiburg Visual Acuity Test^28^. All participants provided written informed consent prior to the study and received financial compensation for their participation. The study was conducted in accordance with the Declaration of Helsinki and was approved by the Ethics Committee of the University Medical Center Freiburg (276/15).

### Study design

**Fig. 1:**
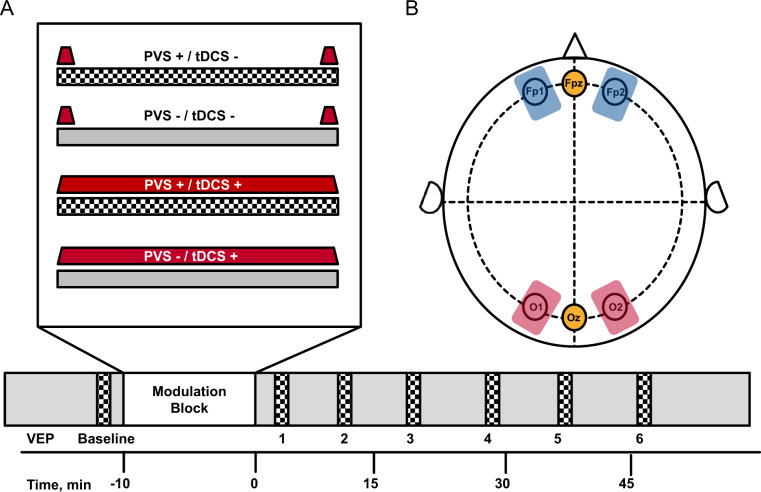
Study design and electrode placement. **A** Study design. Four different experimental conditions were used to modulate visually evoked potentials (VEPs), comprising a combination of either verum (+) or sham stimulation (−) for prolonged visual stimulation (PVS) and transcranial direct current stimulation (tDCS). Every participant performed the experiment over at least two sessions (PVS+/tDCS– and PVS+/tDCS+). Twenty-five participants underwent an additional test session (PVS−/tDCS+), and nine participants were randomized into the double sham control condition (PVS−/tDCS−). Baseline VEP recordings were conducted 2min prior to the stimulation block and experimental VEP recordings 4, 11, 22, 29, 40, and 47min after the stimulation block. **B** Electrode placement. Bioccipital anodal target electrodes were placed over the occipital cortex (O1 and O2, according to the 10–20 electrode positioning system; red), with cathodal return electrodes on the forehead (Fp1 and Fp2; blue). VEPs were recorded from the occipital region on the scalp (Oz; yellow) using a single active gold-cup electrode with a reference electrode placed on the forehead (Fpz; yellow).

One experimental session lasted ~60 min and consisted of baseline VEP induction, one stimulation block and six test VEP measurements. Baseline VEP recordings were conducted 3 min prior to the stimulation block and experimental VEP recordings 4, 11, 22, 29, 40, and 47 min after the stimulation block (Fig. 1A).

The session schedule for all four experimental conditions was identical; the only difference was whether participants received tDCS, prolonged visual stimulation (PVS), either combined or neither during the stimulation block. Every participant performed the experiment for at least two sessions (PVS+/tDCS– and PVS+/tDCS+). Twenty-five participants underwent an additional test session (PVS−/tDCS+), and nine participants out of these 25 received an additional double sham control (PVS−/tDCS−). All sessions were separated by seven days to prevent carry-over effects. Participants were blinded to the tDCS stimulation, with the condition order being counterbalanced across all participants to prevent sequencing effects.

### VEP recordings

The study used the same experimental setup for VEP recordings as previously described by Normann et al.^5^, based on the EP-2000 software (michaelbach.de/ep2000). Stimuli were presented on a CRT screen (Philips GD402) with a resolution of 800 × 600 pixels at a refresh rate of 75 Hz. The monitor was distanced 1.14 m from the participant with mean luminance set to 45 cd/m^2^. Participants were asked to fixate on a target (Ø 0.6°) in the center of the screen and to read the numbers presented at that target at random time intervals out loud to maintain attention during the recording session. To evoke VEPs, a checkerboard reversal pattern inverting twice per second (check size 0.5°) was generated using EP-2000 software (michaelbach.de/ep2000). This reversal frequency was chosen because it produced maximum effect sizes in previous experiments testing several differing frequencies^5^. Between checkerboard stimulation trials, a homogenous gray screen with the same average luminance as the checkerboard was presented, and again, the subjects were asked to read the displayed numbers out loud. Forty checkerboard presentation sweeps were recorded and averaged within 20 s. All trials exceeding 130 μV were considered to be blink artifacts, were excluded, and were replaced by additional sweeps.

Electroencephalographic signals were recorded from the occipital region on the scalp (Oz, according to the 10-10 electrode positioning system) using a single active gold-cup electrode with a reference electrode placed on the forehead (Fpz, according to the 10-10 electrode positioning system; Fig. 1B) and a ground electrode at the left earlobe. Signals were amplified with bandpass filtering of 1 to 100 Hz (electrophysiology amplifier by Roland Consult, Brandenburg an der Havel, Germany), digitizing at 1 kHz. After storage on a computer disk, signals were averaged for offline analysis and low-pass filtered at 40 Hz using IGOR Pro (Wavemetrics, Portland, Oregon). Following filtering, the most positive excursion in the time window [85, 140] ms was detected and labeled P1. C1 then was defined as the most negative excursion in the time window [tP1–38, tP1] ms, and N1 as the most negative excursion in the time window [tP1, tP1 + 60] ms. P1 was identified at 95.5 +/− 9.1 ms, C1 at 71.0 +/− 8.0 ms and N1 at 136.0 +/− 15.0 ms. To further examine a proposed overall measure of VEP plasticity, we analyzed the P1N1 peak-to-peak difference by adding amplitude vectors of P1 and N1. While being slightly redundant in comparison to P1 and N1 single peak analysis, this marker showed the most robust plastic effect in response to PVS^7^.

### Experimental interventions

#### Electrical stimulation

Transcranial direct current stimulation was delivered by a battery-driven, microprocessor-controlled CE-certified constant current stimulator (DC-STIMULATOR PLUS, NeuroConn GmbH, Ilmenau, Germany). Bioccipital anodal target rubber electrodes covered with conductive electrode cream (Ten20® Conductive Paste, Weaver and company) were placed over the occipital cortex (O1 and O2, according to the 10–20 electrode positioning system; 5 × 7 cm; Fig. 1B), with cathodal return electrodes on the forehead (Fp1 and Fp2; 5 × 7 cm). Prior to stimulation, the skin was cleaned with 70% v/v isopropyl alcohol and abrasive gel (NuPrep Abrasive Skin Prep gel, Weaver and company). For robust effects within the safety recommendations, a constant current of 1 mA was applied over each electrode (2 mA stimulator output, Y-cable split for target and return electrodes; duration 10 min total; tDCS+)^29^. A fade-in/fade-out design (30 s each) was used to decrease potential skin sensations during the beginning and end of the stimulation^17^. In the sham condition, a 30 s fade-in was immediately followed by 30 s fade-out at the beginning and the end of the stimulation block without active stimulation in between (10 min total; tDCS−). This sham procedure has repeatedly been reported to keep the participants blinded to the stimulation conditions^30,31^. In the current study, participants generally overestimated the application of an active tDCS stimulation condition (81.0% of all time points). Only 27.9% of sham stimulations were correctly labeled as such by the participants proving sufficient blinding for the conditions.

#### Prolonged visual stimulation

To provoke VEP plasticity, the described standard checkerboard reversal pattern (PVS+) was presented for a total duration of 10 min with presentation of the homogenous gray screen (PVS−) as the control condition.

### Data analysis

Artifact-free trials were averaged according to the stimulation condition and time point. VEP amplitudes from all six poststimulation recordings were then averaged as poststimulation VEPs. Peak amplitudes of the VEP components C1, P1, and N1, as well as P1N1, were measured in component direction for better comparability (i.e., amplitudes of negative-going components are positive) and used for statistical testing performed in SPSS Statistics (IBM SPSS Statistics for Windows, IBM Corp., Armonk, NY). Descriptive values are given as means and standard deviations. For the estimation of effect sizes, partial *η* square (p *η*^2^) values were calculated (low: <0.06; medium: ≥0.06 and <0.14; large: ≥0.14). The level of significance was set at *p* < 0.05 (two-tailed).

The main analysis was based upon component amplitude changes computed as differences between mean postintervention amplitudes and baseline amplitudes for each component. As the different experimental conditions were completed by different subsets of participants, linear mixed effects models were used for the detection of generalized stimulation effects. In a linear mixed effects model regression coefficients represent estimates of otherwise unknown population parameters and describe the relationship between two variables (predictor variable and response variable). For example, in our case the effect of “no stimulation” on VEP plasticity was estimated by including data from each stimulation condition that comprised at least one sham stimulation paradigm (PVS−/tDCS+; PVS+/tDCS−, and PVS−/tDCS−) to the linear mixed effects model. The coefficient values for each stimulation type might therefore be slightly different to the measured values in the corresponding stimulation condition. In addition to analyzing those model coefficients for the capability of both stimulation types to induce VEP plasticity in comparison to the estimated VEP component amplitude change without intervention, each linear mixed effects model was analyzed for each VEP component separately using analysis of variance (ANOVA) *F*-test statistic with the within-subject factors PVS (+/−) and tDCS (+/−).

In an exploratory approach, VEP plasticity was then assessed with repeated measures ANOVA with the within-subject factor Time (baseline, poststimulation) for each VEP component and each stimulation condition separately. The percentage of VEP amplitude change was estimated to visualize the size of the stimulation effects. To delineate the time course of the stimulation effects, all six poststimulation VEPs were then analyzed separately with repeated measures ANOVA *F*-test statistic with the within-subject factor Time (baseline, poststimulation VEP 1–6) for every condition.

## Results

To analyze main effects of either tDCS or PVS on VEP plasticity, we implemented linear mixed effects models including data from all four conditions and analyzed the pre-post-stimulation-difference of each VEP component separately using ANOVA with the within-subject factors PVS (+/−) and tDCS (+/−). The linear model showed that C1 modulation was larger with tDCS (coefficient: −3.6 μV ± 7.9 μV [SD]; *p* < 0.001; *d* = −0.46) than with PVS (coefficient: −2.3 μV ± 7.6 μV [SD]; *p* = 0.014; *d* = −0.30), or both (coefficient: +3.5 μV ± 9.1 μV [SD]; *p* = 0.002; *d* = 0.38), but all stimulation types led to an amplitude decrease instead of an increase as estimated for the no stimulation control condition (coefficient: +1.6 μV ± 7.0 μV [SD]). Correspondingly for C1, ANOVA detected a significant influence of tDCS (*F* = 4.3; *p* = 0.04) and a significant interaction of between both stimulations (*F* = 10.1; *p* = 0.002). For P1, no significant differences could be detected. All stimulation conditions led to a slight amplitude increase comparable to the estimated control condition (coefficient: +1.1 μV ± 7.0 μV [SD]; all *p* > 0.05). For N1, a differing modulation effect was found for PVS (coefficient: −1.6 μV ± 6.1 μV [SD]; *p* = 0.035; *d* = −0.26) and tDCS (coefficient: −2.1 μV ± 6.3 μV [SD]; *p* = 0.006; *d* = −0.34), as well as for both (coefficient: +1.9 μV ± 7.2 μV [SD]; *p* = 0.034, *d* = 0.26) compared to the estimated “no stimulation” condition that led again to a relevant amplitude increase (coefficient: +1.9 μV ± 5.7 μV [SD]). Correspondingly, ANOVA detected a significant interaction between stimulation types (*F* = 4.7; *p* = 0.034), with tDCS showing an effect at trend level (*F* = 3.1, *p* = 0.084). Analyzing the P1N1 amplitude range further substantiated the effects by demonstrating an almost significant difference for PVS (−1.6 μV ± 6.6 μV [SD]; *p* = 0.055, *d* = −0.24) and significant differences for tDCS (−1.9 μV ± 6.8 μV [SD]; *p* = 0.026; *d* = 0.28), as well as for both (1.9 μV ± 7.6 μV [SD]; *p* = 0.048, *d* = 0.24) compared no stimulation (3.1 μV ± 6.3 μV [SD]). ANOVA again detected a significant interaction between tDCS and PVS (*F* = 4.0, *p* = 0.049).

**Fig. 2:**
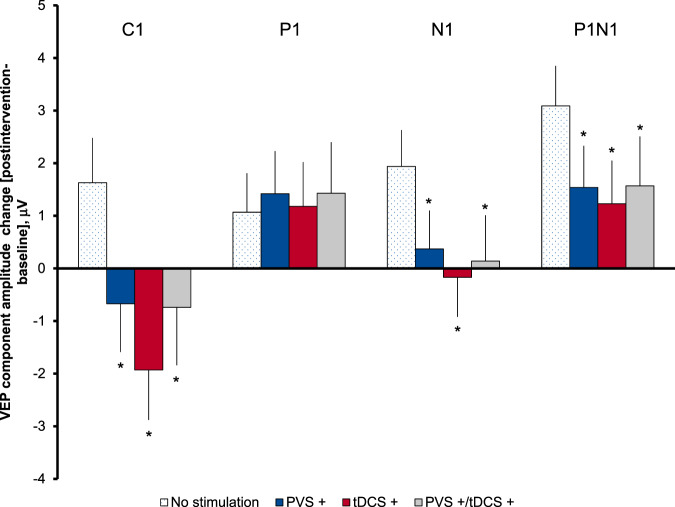
Postintervention VEP component amplitude change [postintervention-baseline] depending on condition. All amplitudes were measured in component direction for better comparability (i.e., amplitudes of negative-going components are positive). It is to note, that the estimated coefficients as given by the linear mixed effects models need to be added to each other to get the overall estimation of effect sizes for a specific stimulation type. For example, for the stimulation condition PVS, the respective coefficient for PVS+ needs to be added to the coefficient for “no stimulation.” To correctly display the influence of combined stimulation, the coefficients of “no stimulation”, PVS+, tDCS+, and the additional coefficient for the PVS/tDCS interaction needs to be summed up. While all VEP components are estimated to slightly increase in the absence of stimulation in the linear mixed effects models, both experimental conditions displayed significantly different effects. Mainly, C1 amplitudes decreased after PVS, tDCS, and combined stimulation. N1 increased, but to a significantly smaller degree than without stimulation after PVS and combined stimulation and decreased following tDCS. P1N1 peak-to-peak difference increased following all conditions, but to a significantly smaller amount following tDCS and combined stimulation than estimated for the absence of stimulation. Combined stimulation in general displayed effect seizes similar to the single stimulation conditions and did not reach levels as expected for additive effects. VEP, visually evoked potential; PVS+, sum of coefficients for prolonged visual stimulation and “no stimulation”; tDCS+, sum of coefficients for transcranial direct current stimulation and “no stimulation”, PVS+/tDCS+, sum of coefficients for PVS+, tDCS+, PVS/tDCS interaction and “no stimulation”; β-coefficients ± SE. Asterisk indicates significant difference in amplitude changes following the stimulation condition compared to “no stimulation” (significant coefficient in linear mixed effects model).

The resulting total effect of each stimulation type on VEP component amplitude change between baseline and postintervention measurement are visualized in Fig. 2. First, the value for the “no stimulation” condition as estimated by the model is displayed. For the following stimulation conditions PVS+ and tDCS+, the respective coefficients are added to the coefficient for “no stimulation.” To correctly display the influence of combined stimulation, the coefficients of “no stimulation”, PVS+, tDCS+ and the additional coefficient for the PVS/tDCS interaction are summed up (Fig. 2). In summary, combined stimulation displayed effect sizes similar to the single stimulation conditions for all VEP components but did not reach levels as expected for additive effects.

To further examine these effects on VEP plasticity, all conditions were then analyzed in detail separately in an exploratory approach.

### Induction of VEP plasticity by prolonged visual stimulation

**Fig. 3:**
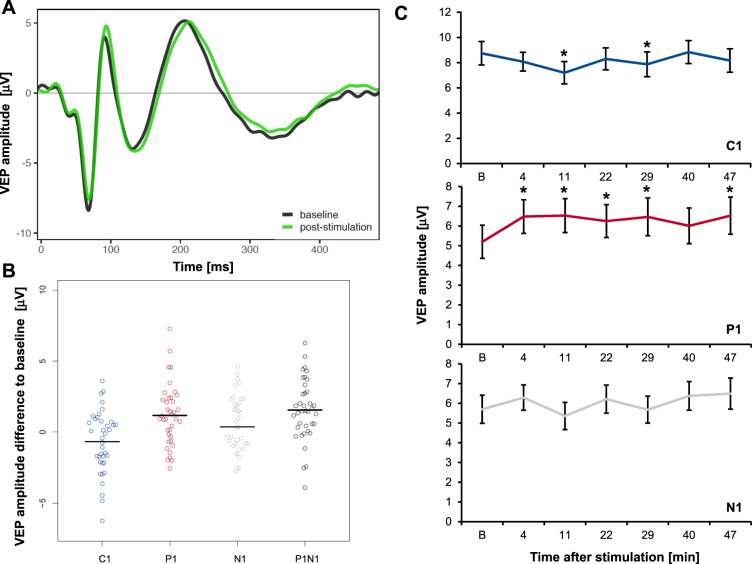
Prolonged visual stimulation induced VEP plasticity (*n* = 38, PVS+/tDCS−). **A** Checkerboard reversal VEP traces before (black) and averaged poststimulation VEPs (green) after the presentation of a 10-min checkerboard reversal stimulation block. **B** Change in peak VEP amplitudes averaged after stimulation compared to baseline (horizontal line, mean). P1 and P1N1 increased significantly. **C** Mean peak amplitudes of C1, P1, and N1 VEP components over time +/− SE. Asterisk indicates significant post hoc contrast of baseline peak amplitude [B] compared to test peak amplitude. VEP visually evoked potential, PVS prolonged visual stimulation, tDCS transcranial direct current stimulation.

First, to specify whether PVS without tDCS (PVS+/tDCS−) resulted in the plastic modulation of early VEP components (Fig. 3A), each peak was analyzed separately (Fig. 3B). While C1 demonstrated a slight decrease of 7.7%, almost reaching statistical significance (*F* = 3.6; *p* = 0.067; p *η²* = 0.088), P1 significantly increased by 24.8% (*F* = 11.3; *p* = 0.002; p *η²* = 0.233). N1 increased by 6.5% but showed a very high variance, leading to no statistically detectable effect (*F* = 0.9; *p* = 0.339; p *η²* = 0.025). To further examine a proposed overall measure of VEP plasticity^7^, we analyzed the P1N1 peak-to-peak difference, which showed a highly significant increase of 14.2% following PVS (*F* = 19.3; *p* = <0.001; p *η²* = 0.343).

To explore the time course of VEP plasticity induced by PVS, VEP peak amplitudes from all single time points after stimulation were separately compared with baseline peak amplitudes. For C1, we discerned a transient amplitude reduction 11 to 29 min after stimulation, while for P1, we detected a sustained amplitude increase for at least 50 min (Fig. 3C and Table S1, supplements).

### No induction of VEP plasticity by short visual stimulation

As described in the methods section, the same checkerboard reversal rate for VEP recording was used during the test blocks and PVS in the stimulation block (test block: 40 reversals within 20 s; stimulation block: 1200 reversals within 10 min). To control for the putative plastic effects related to the test blocks alone, a homogenous gray screen was presented for 10 min instead of PVS stimulation in 9 participants (PVS−/tDCS−). No significant modulation of VEP amplitudes was detected in these participants (C1: *F* = 0.9; *p* = 0.358; p *η²* = 0.106; P1: *F* = 0.9; *p* = 0.360; p *η²* = 0.105; N1: *F* = 3.7; *p* = 0.090; p *η²* = 0.317, supplemental Fig. S2). This finding supports that VEP plasticity depends on sufficient and specific stimulation.

### Induction of VEP plasticity by bioccipital anodal tDCS

**Fig. 4:**
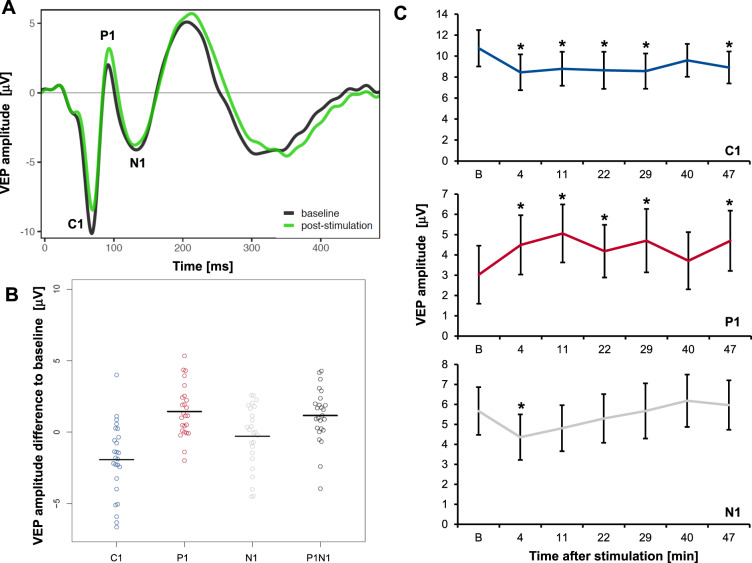
tDCS-induced VEP plasticity (*n* = 25, PVS−/tDCS+). **A** Checkerboard reversal VEP traces before (black) and averaged poststimulation VEPs (green) after 10 min of occipital anodal tDCS. **B** Change in peak VEP amplitudes averaged after stimulation compared to baseline (horizontal line, mean). C1 decreased and P1 and P1N1 increased significantly. **C** Mean peak amplitudes of C1, P1, and N1 VEP components over time +/− SE. Asterisk indicates significant post hoc contrast of baseline peak amplitude [B] compared to test peak amplitude. VEP visually evoked potential, PVS prolonged visual stimulation, tDCS transcranial direct current stimulation.

To examine direct tDCS effects on VEP plasticity, 25 participants received bioccipital anodal tDCS without additional prolonged visual stimulation (PVS−/tDCS+). The stimulation paradigm resulted in the plastic modulation of early VEP components (Fig. 4A). While C1 demonstrated a significant decrease of 17.9% (*F* = 14.0; *p* = 0.001; p *η²* = 0.368), P1 significantly increased by 47.9% (*F* = 16.0; *p* = 0.001; p *η²* = 0.339), and the P1N1 peak-to-peak difference increased by 13.4% (*F* = 9.5; *p* = 0.005; p *η²* = 0.284). N1 slightly decreased by 5.1% without reaching significance (*F* = 0.4; *p* = 0.514; p *η²* = 0.018; Fig. 4B). Regarding the time course of the modulation by tDCS, we found a stable amplitude modulation for both C1 and P1 over at least 50 min. N1 amplitudes were transiently reduced immediately after stimulation but were quickly restored to baseline levels (Fig. 4C and Table S1, supplements).

### Induction of VEP plasticity by combined prolonged visual stimulation and tDCS

**Fig. 5:**
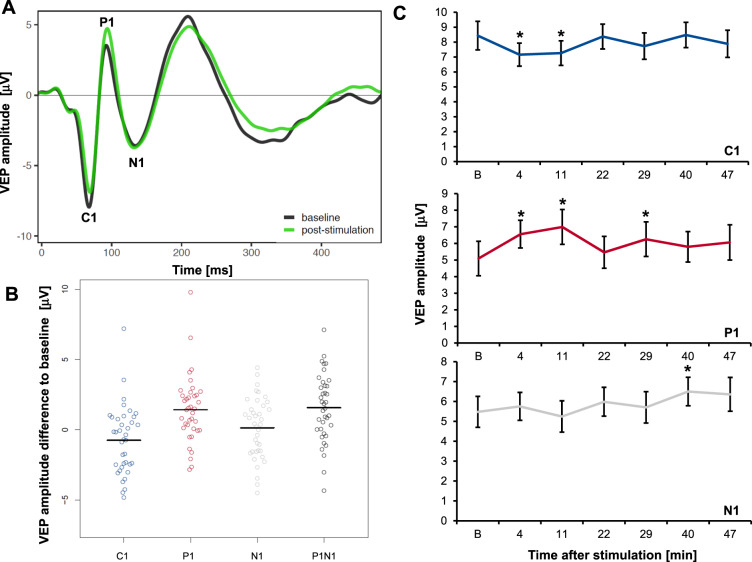
Combined tDCS- and PVS-induced VEP plasticity (*n* = 38, PVS+/tDCS+). **A** Checkerboard reversal VEP traces before (black) and averaged poststimulation VEPs (green) after 10 min of occipital anodal tDCS parallel to the presentation of a 10-min checkerboard reversal stimulation block. **B** Change in peak VEP amplitudes averaged after stimulation compared to baseline peak amplitude (horizontal line, mean). P1 and P1N1 increased significantly. **C** Mean peak amplitudes of C1, P1, and N1 VEP components over time +/− SE. Asterisk indicates significant post hoc contrast of baseline peak amplitude [B] compared to test peak amplitude. VEP visually evoked potential, PVS prolonged visual stimulation, tDCS transcranial direct current stimulation.

To investigate whether a combination of PVS and tDCS changed the overall effects on VEP plasticity, all 38 participants received combined PVS and occipital anodal tDCS (PVS+/tDCS+). The stimulation paradigm resulted in a plastic modulation of early VEP components (Fig. 5A). Looking at each peak separately (Fig. 5B), C1 demonstrated a slight, but not significant decrease of 7.3% (*F* = 2.3; *p* = 0.136; p *η²* = 0.059), P1 significantly increased by 21.5% (*F* = 6.3; *p* = 0.017; p *η²* = 0.145), and N1 increased by 8.2% without statistical significance (*F* = 1.3; *p* = 0.259; p *η²* = 0.034). The P1N1 peak-to-peak difference displayed a highly significant increase of 14.6% (*F* = 14.0; *p* = 0.001; p *η²* = 0.275). The decrease in C1, as well as the increase in P1, amplitudes compared to baseline VEP amplitude values was time-limited to 11 to 29 min following combined stimulation (Fig. 5C and Table S1, supplements).

## Discussion

In this study, we combined prolonged visual stimulation (PVS), as an established model to induce and analyze VEP plasticity in the human brain, and concurrent tDCS stimulation for the first time. We demonstrated the induction of VEP plasticity by tDCS, which was comparable in magnitude and time course to VEP plasticity induced by PVS. Both stimulation types decreased amplitudes of C1 compared to no stimulation and led to a significantly smaller increase (PVS) or even decrease (tDCS) in N1 amplitudes compared to no stimulation. The P1N1 peak-to-peak difference increased following all stimulation types, but to a significantly lesser degree for active stimulation, than what was estimated for no stimulation. While all stimulation types increased P1 amplitudes, the linear mixed effects model did not detect a significant difference between active stimulation and no stimulation. Combined stimulation induced sustained plastic modulation of C1 and N1 but with a smaller effect size than what would be expected for an additive effect.

To fully understand the data, we then analyzed each stimulation condition separately to describe changes between pre- and poststimulation VEP measurements and the respective time course of effects. By using the protocol of Normann et al.^5^, we replicated prior findings and found that PVS induces sustained VEP plasticity. Specifically, the amplitude of P1, as well as the P1N1 peak-to-peak difference, was significantly increased after PVS compared to baseline. This VEP plasticity lasted for a minimum of 50 min after stimulation. Replicating prior experiments, we confirmed that the limited visual stimulation for recording VEPs during the test epochs in the absence of prolonged visual or electric stimulation did not cause changes in VEP plasticity.

Bioccipital anodal tDCS-induced sustained VEP plasticity. Importantly, we demonstrated that tDCS produced measurable, reliable and specific responses in the brain, refuting the aforementioned uncertainties^24^. The amplitude of P1 and P1N1 were significantly increased, while the C1 amplitude was significantly decreased. These induced effects lasted until the conclusion of the experiment, 50 min after stimulation. Only a few studies have examined tDCS effects in the visual cortex. Antal et al.^22^ tested three different electrode montages and stimulation durations between 5 and 15 min. This group only found tDCS effects at a setting similar to the one used in the present study but only for one electrode pairing (Oz-Cz). While anodal stimulation failed to induce a significant modulatory effect, cathodal stimulation led to a reduction in C1 amplitudes and a tendency to increase P1^22^. The authors suggested that the lack of effects of anodal stimulation could have been attributed to insufficient stimulation strength. The more robust effects of anodal stimulation in the present experiments could be explained by the slightly different electrode montage, the increased total electrical current (2 mA) and the bilateral stimulation paradigm. The reliability of our results is further supported by the similarities of the demonstrated aftereffects between the motor and visual cortex. Most studies on tDCS-induced plasticity in the motor cortex demonstrated similarly long aftereffects of 30–60 min following sufficient stimulation of ~10 min^29,32^.

To examine whether concurrent tDCS and PVS induce metaplastic effects, we applied both stimulation modalities at the same time. A combined visual and electric stimulation protocol induced sustained VEP plasticity, with a significant interaction detected for C1 and N1 peaks in the linear mixed effects model. The β-coefficients imply no additive or metaplastic effect but rather displayed a smaller effect size than expected for a strictly additive effect of combined PVS and tDCS (Fig. 2). This result might represent a ceiling effect for interventions that share a common electrophysiological mechanism. The lack of metaplastic effects from tDCS, as conceptualized by some authors^19^, might additionally be related to the time course of the present experiment. Most studies on tDCS demonstrate the importance of timing for detecting specific tDCS effects and propose applying tDCS either concurrently with^33^ or prior to^19^ a plasticity-dependent intervention. Future studies should test whether applying tDCS prior to PVS induces a detectable metaplastic shift in VEP plasticity.

The present results demonstrate, for the first time, the direct induction of LTP-like plasticity resulting in distinct and specific changes in VEP components by anodal tDCS in the human visual cortex. These findings strongly suggest that tDCS is able to directly induce plastic LTP-like effects in the human cortex.

The findings should be interpreted in the light of the following limitations: to demonstrate reliable effects within a feasible group size, the present study sample was restricted to young adults within a relatively narrow age range. As a decline in LTP inducibility has been proposed for higher ages^34^, generalization of the results might be limited in the elderly. In addition, it remains unclear whether cathodal stimulation, using the same or differing experimental settings^35^, might induce similar effects and how important the polarity of the electric field is in regards to VEP plasticity.

It is to note that the study tested only a subgroup of nine participants for the double placebo condition receiving neither PVS nor tDCS. While the lack of VEP plasticity induction in the absence of stimulation is well known^5^, the different sample sizes might have led to an increased influence of the single stimulation conditions (PVS+, tDCS+) on the interpretation of the proposed time course for “no stimulation” in the linear mixed effects models. This might explain the similar P1 amplitude change for active and no stimulation in the model, as well as the slight difference to the effects as examined for each experimental condition alone.

In the motor cortex, the application of anodal tDCS enhanced the amplitude of low-frequency motor-evoked potentials (MEP); this effect was *N*-methyl-d-aspartate (NMDA)-receptor-dependent^20^. In a mouse model, tDCS-induced brain-derived neurotrophic factor (BDNF)- and NMDA-dependent long-lasting synaptic potentiation in brain slices from the motor cortex when applied during repetitive low-frequency synaptic stimulation^21^. These and other findings suggest that tDCS-induced plasticity and LTP in brain slices share common mechanisms^36^. Taken together, both data from animal and human research suggest that tDCS induces an LTP-like process in cortical synapses. In the current study, we show that, on an intraindividual level, electrical tDCS stimulation over the occipital cortex is able to replace PVS for the induction of VEP plasticity. VEP plasticity, as a form of stimulus-selective response plasticity, has been conceptualized both in animal models and in human studies as a naturally occurring correlate of LTP in the brain^37^. We therefore propose that anodal tDCS induces LTP in the human cortex.

Impaired LTP-like plasticity has been hypothesized to be a common underlying mechanism of various mental disorders, such as depression^5,25,26,38^, dementia^39^, and schizophrenia^40,41^. In major depression, both data from human and animal research conclusively suggest a dysregulation of synaptic plasticity, which can be corrected by antidepressive treatment^5,25,26,42–44^. tDCS is therefore considered a promising therapeutic tool for these disorders, both by the putative direct restoration of disturbed synaptic plasticity^45^ and by the augmentation of other treatment modalities such as medication^46,47^ and psychotherapy^14,48^. Plasticity changes induced by tDCS might be related to very basic and ubiquitous mechanisms, suggesting that our findings could be generalized to different stimulation localizations and modalities. This hypothesis is supported by experimental evidence of an intraindividual correlation between the amount of plasticity induced by rTMS in the motor cortex and by VEP plasticity in the visual cortex^49^.

Relatively modest effects of current therapeutic tDCS protocols^50,51^ suggest the need for the development of optimized treatment modalities^24,52^. The results of the current study add to the evidence that tDCS is capable of directly increasing LTP-like plasticity in the human brain and aid in further implementing VEP plasticity in the visual cortex as a potential tool to evaluate the neuromodulatory effects of treatment approaches in the context of mental disorders.

## Supplementary information

Table S1

Figure S1

Figure S2
