# Supplementary material for: Transcranial direct current stimulation induces long-term potentiation-like plasticity in the human visual cortex
Source: Transl Psychiatry. 2021 Jan 4;11:17. doi: 10.1038/s41398-020-01134-4 (PMC7791098; doi:10.1038/s41398-020-01134-4)
Supplement: Supplementary file 1 — Table S1 [file 41398_2020_1134_MOESM1_ESM.docx]

|  |  | **T1** | | | **T2** | | | **T3** | | | **T4** | | | **T5** | | | **T6** | | |
| --- | --- | --- | --- | --- | --- | --- | --- | --- | --- | --- | --- | --- | --- | --- | --- | --- | --- | --- | --- |
| **Condition** | **Peak** | Δ μV | *p* | p *η²* | Δ μV | *p* | p *η²* | Δ μV | *p* | p *η²* | Δ μV | *p* | p *η²* | Δ μV | *p* | p *η²* | Δ μV | *p* | p *η²* |
| **PVS +/tDCS -** | C1 | -.67 | .120 | .064 | -1.55 | **.002** | .227 | -.45 | .374 | .021 | -.88 | **.041** | .108 | .09 | .831 | .001 | -.58 | .208 | .043 |
| **(N=38)** | P1 | 1.28 | **.015** | .150 | 1.33 | **.017** | .144 | 1.05 | **.009** | .169 | 1.26 | **.017** | .144 | 0.81 | .052 | .098 | 1.33 | **.005** | .191 |
|  | N1 | .59 | .208 | .043 | -.34 | .535 | .010 | .52 | .196 | .045 | -.02 | .966 | <.001 | .68 | .117 | .065 | .79 | .161 | .053 |
| **PVS -/tDCS +** | C1 | -2.30 | **.005** | .289 | -1.96 | **.004** | .291 | -2.10 | **.008** | .256 | -2.18 | **.002** | .328 | -1.16 | .061 | .139 | -1.84 | **.007** | .265 |
| **(N=25)** | P1 | 1.47 | **.009** | .255 | 2.03 | **<.001** | .407 | 1.16 | **.040** | .165 | 1.68 | **.013** | .232 | .69 | .126 | .095 | 1.67 | **.002** | .325 |
|  | N1 | -1.31 | **.017** | .215 | -.86 | .174 | .076 | -.37 | .513 | .018 | .01 | .993 | <.001 | .52 | .366 | .034 | .30 | .650 | .009 |
| **PVS +/tDCS +** | C1 | -1.28 | **.013** | .156 | -1.17 | **.026** | .127 | -.06 | .885 | .001 | -.71 | .148 | .056 | .04 | .934 | <.001 | -.55 | .270 | .033 |
| **(N=38)** | P1 | 1.47 | **.016** | .147 | 1.90 | **.002** | .230 | .37 | .485 | .013 | 1.16 | **.016** | .148 | .70 | .162 | .052 | .97 | .066 | .088 |
|  | N1 | .28 | .533 | .011 | -.23 | .627 | .006 | .51 | .274 | .032 | .23 | .669 | .005 | 1.02 | **.042** | .107 | .88 | .087 | .077 |
| **PVS -/tDCS -** | C1 | -.34 | .824 | .007 | 1.12 | .484 | .063 | 2.34 | .239 | .168 | 1.9 | .337 | .115 | 1.58 | .400 | .090 | 2.38 | .237 | .170 |
| **(N=9)** | P1 | .92 | .226 | .177 | 1.03 | .361 | .105 | .28 | .879 | .003 | 2.08 | .168 | .223 | .43 | .689 | .021 | 1.44 | .361 | .105 |
|  | N1 | .20 | .846 | .005 | 1.66 | .158 | .232 | 2.14 | **.012** | .567 | .39 | .687 | .021 | 2.70 | **.042** | .423 | 1.34 | .192 | .203 |

Uncorrected ANOVAs with the within factor Time (pre, post stimulation [mean]). PVS, prolonged visual stimulation; tDCS, transcranial direct current stimulation; Δ μV, amplitude change in μV (post intervention time point T1-6 – baseline);p η², partial eta square. Significant effects are marked as bold.
