# Supplementary material for: Transcranial direct current stimulation induces long-term potentiation-like plasticity in the human visual cortex
Source: Transl Psychiatry. 2021 Jan 4;11:17. doi: 10.1038/s41398-020-01134-4 (PMC7791098; doi:10.1038/s41398-020-01134-4)
Supplement: Supplementary file 2 — Figure S1 [file 41398_2020_1134_MOESM2_ESM.pptx]

## Slide 1
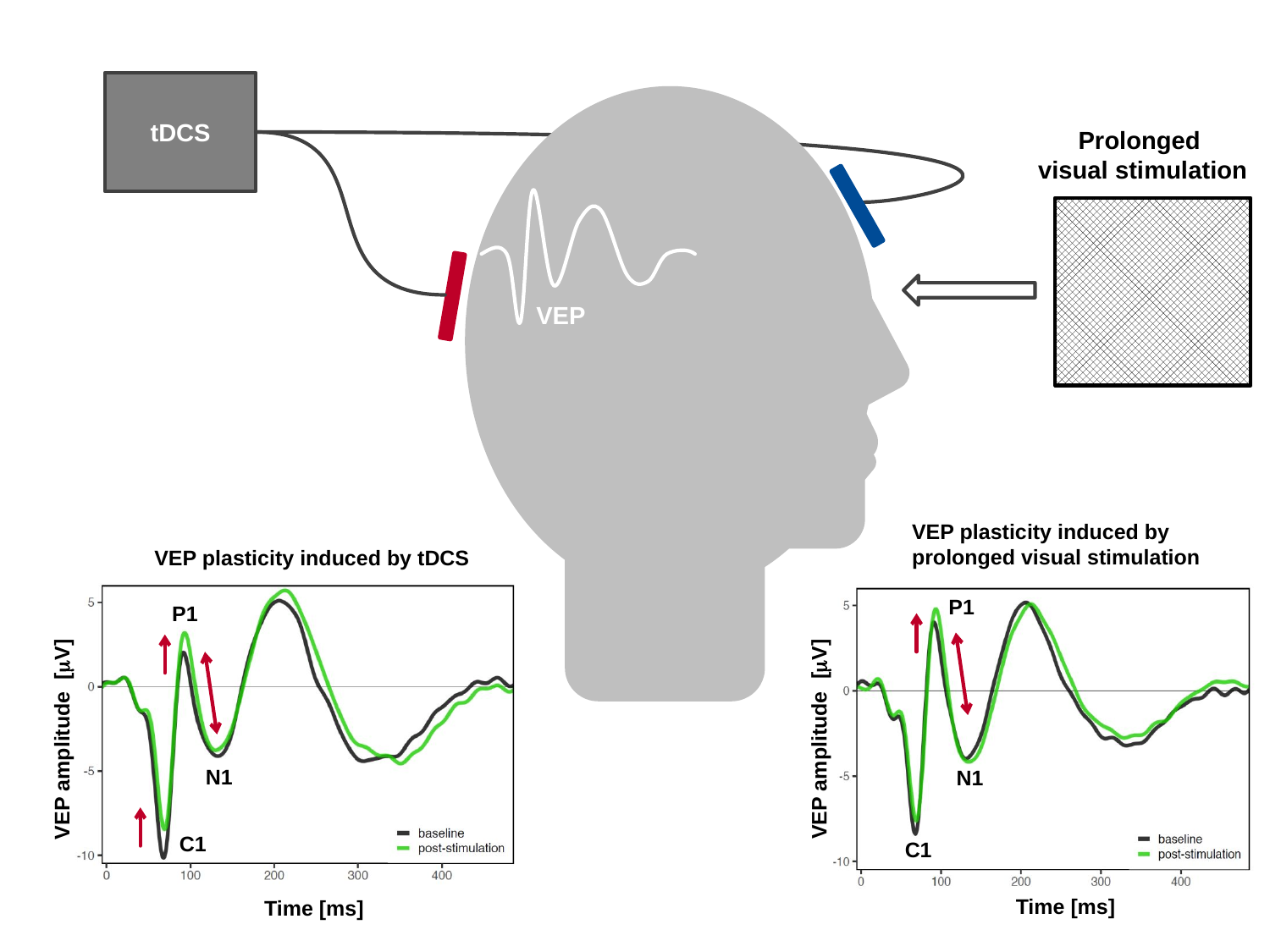

tDCS
Prolonged
visual stimulation
VEP
a
VEP plasticity induced by
prolonged visual stimulation
VEP plasticity induced by tDCS
VEP amplitude [mV]
Time [ms]
P1
P1
VEP amplitude [mV]
N1
N1
C1
C1
Time [ms]
