# Supplementary material for: Transcranial direct current stimulation induces long-term potentiation-like plasticity in the human visual cortex
Source: Transl Psychiatry. 2021 Jan 4;11:17. doi: 10.1038/s41398-020-01134-4 (PMC7791098; doi:10.1038/s41398-020-01134-4)
Supplement: Supplementary file 3 — Figure S2 [file 41398_2020_1134_MOESM3_ESM.pptx]

## Slide 1
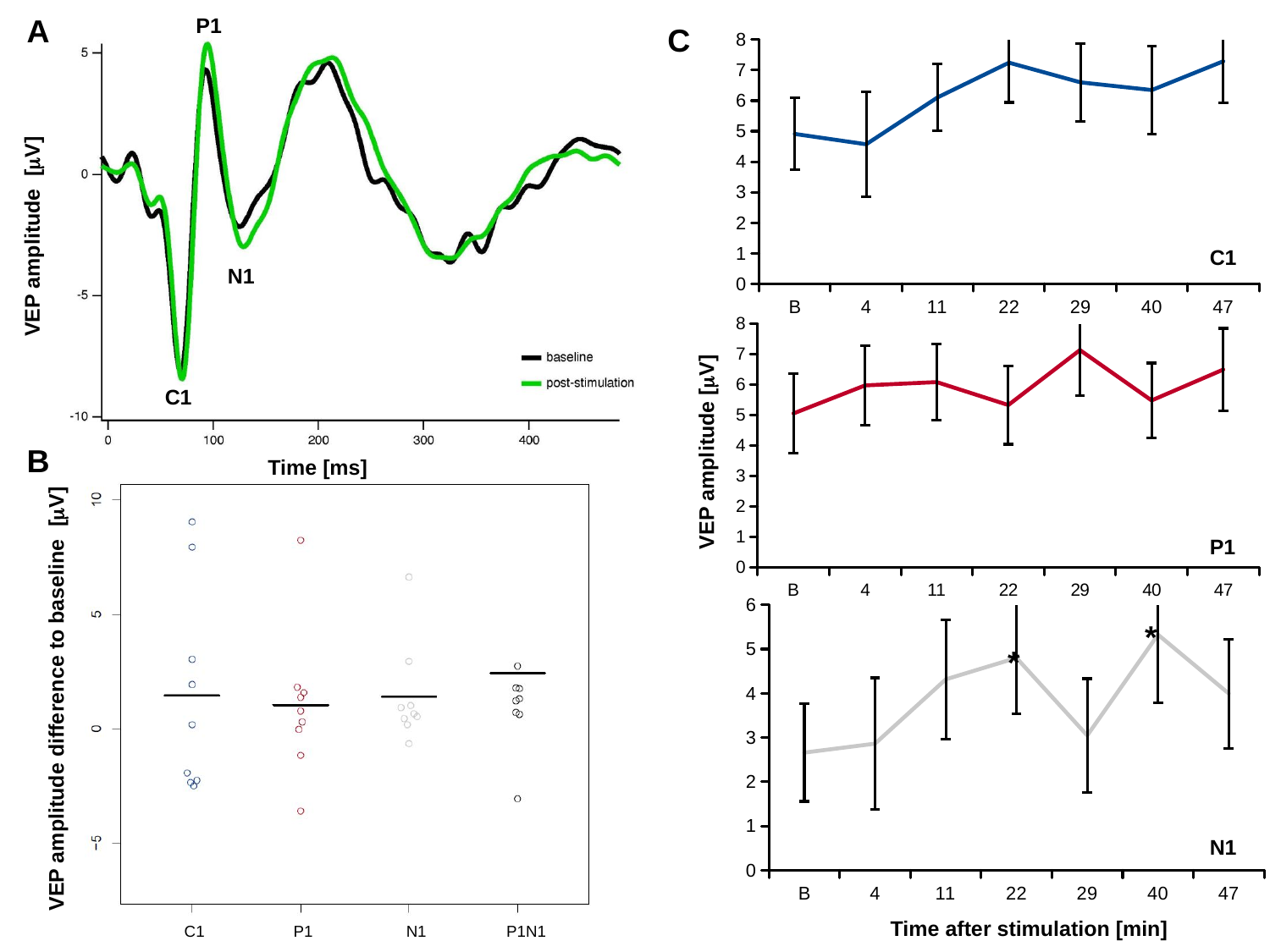

A
P1
C
### Chart
| Category | Mittelwert |
|---|---|
| B | 4.91 |
| 4 | 4.571111111111112 |
| 11 | 6.1066666666666665 |
| 22 | 7.245555555555554 |
| 29 | 6.6 |
| 40 | 6.348888888888889 |
| 47 | 7.29111111111111 |VEP amplitude [mV]
C1
N1
### Chart
| Category | Mittelwert |
|---|---|
| B | 5.062222222222222 |
| 4 | 5.982222222222222 |
| 11 | 6.090000000000001 |
| 22 | 5.336666666666667 |
| 29 | 7.142222222222223 |
| 40 | 5.488888888888889 |
| 47 | 6.501111111111111 |C1
VEP amplitude [mV]
B
Time [ms]
P1
### Chart
| Category | Mittelwert |
|---|---|
| B | 2.658888888888889 |
| 4 | 2.8622222222222224 |
| 11 | 4.315555555555555 |
| 22 | 4.800000000000001 |
| 29 | 3.0477777777777777 |
| 40 | 5.325555555555556 |
| 47 | 3.9944444444444445 |*
*
VEP amplitude difference to baseline [mV]
N1
Time after stimulation [min]
C1 P1 N1 P1N1

## Slide 2
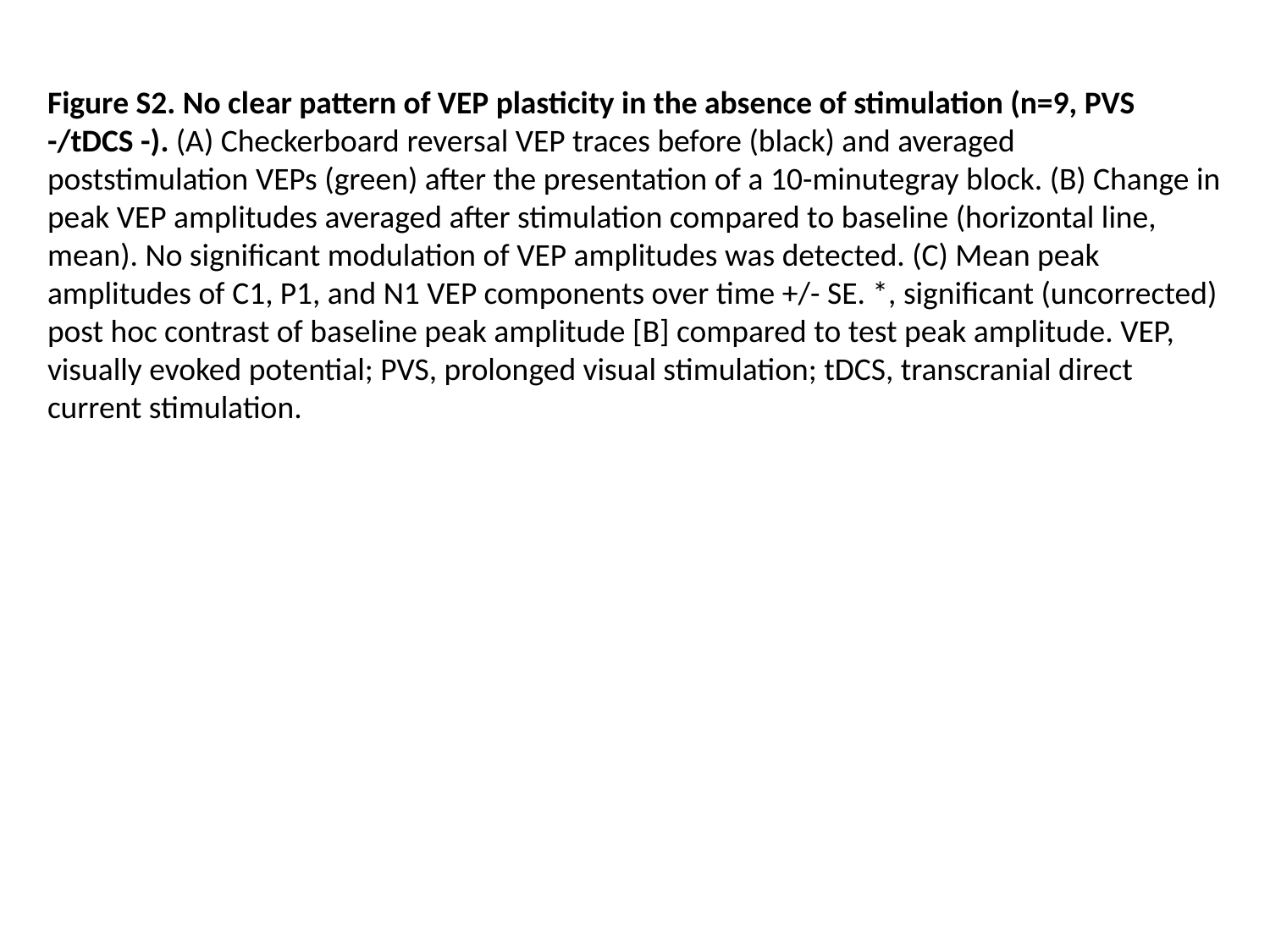

Figure S2. No clear pattern of VEP plasticity in the absence of stimulation (n=9, PVS -/tDCS -). (A) Checkerboard reversal VEP traces before (black) and averaged poststimulation VEPs (green) after the presentation of a 10-minutegray block. (B) Change in peak VEP amplitudes averaged after stimulation compared to baseline (horizontal line, mean). No significant modulation of VEP amplitudes was detected. (C) Mean peak amplitudes of C1, P1, and N1 VEP components over time +/- SE. *, significant (uncorrected) post hoc contrast of baseline peak amplitude [B] compared to test peak amplitude. VEP, visually evoked potential; PVS, prolonged visual stimulation; tDCS, transcranial direct current stimulation.
